# The role of angiogenesis in melanoma: Clinical treatments and future expectations

**DOI:** 10.3389/fphar.2022.1028647

**Published:** 2022-12-15

**Authors:** Zhuzhu Wu, Yifei Bian, Tianjiao Chu, Yuman Wang, Shuai Man, Yongmei Song, Zhenguo Wang

**Affiliations:** ^1^ Experimental Center, Shandong University of Traditional Chinese Medicine, Jinan, China; ^2^ Institute for Literature and Culture of Chinese Medicine, Shandong University of Traditional Chinese Medicine, Jinan, China; ^3^ Innovation Research Institute of Traditional Chinese Medicine, Shandong University of Traditional Chinese Medicine, Jinan, China; ^4^ Key Laboratory of Traditional Chinese Medicine for Classical Theory, Ministry of Education, Shandong University of Traditional Chinese Medicine, Jinan, China; ^5^ Shandong Provincial Key Laboratory of Traditional Chinese Medicine for Basic Research, Shandong University of Traditional Chinese Medicine, Jinan, China

**Keywords:** angiogenesis, melanoma, clinical treatment, pharmacology, mechanism

## Abstract

The incidence of melanoma has increased rapidly over the past few decades, with mortality accounting for more than 75% of all skin cancers. The high metastatic potential of Melanoma is an essential factor in its high mortality. Vascular angiogenic system has been proved to be crucial for the metastasis of melanoma. An in-depth understanding of angiogenesis will be of great benefit to melanoma treatment and may promote the development of melanoma therapies. This review summarizes the recent advances and challenges of anti-angiogenic agents, including monoclonal antibodies, tyrosine kinase inhibitors, human recombinant Endostatin, and traditional Chinese herbal medicine. We hope to provide a better understanding of the mechanisms, clinical research progress, and future research directions of melanoma.

## 1 Introduction

Melanoma is one of the most aggressive and fatal skin cancer types, characterized by rapid growth, a long dormancy time, high rates of late-stage recurrence, and extensive metastasis (Eddy and Chen, 2020; Filippi et al., 2020). Its incidence has steadily increased over the past few decades, posing a significant threat to human health worldwide (Li et al., 2022). The considerable risk factor for Melanoma is UV radiation *via* direct DNA damage and harmful effects on the skin (Sample and He, 2018). Acquired and congenital nevus are also risk factors for melanoma (Li et al., 2019a). Approximately 25% of patients with melanoma develop from nevus, and 5%–15% of patients with a family history were susceptible to melanoma (Armstrong and Cust, 2017). Indeed, patients with melanoma who were diagnosed at an early stage could be cured by surgical removal. However, tumor metastasis always occurs after initial treatments and is a fundamental cause of the recurrence in patients with melanoma. Although, clinical therapeutic options for melanoma are plentiful, such as chemotherapy, immunotherapy, and other targeted therapies, the prognosis of advanced melanoma remains severe (Ramelyte et al., 2017; Xiao et al., 2019; Goldinger et al., 2022). Thus, a new and effective therapeutic method is still needed to treat melanoma.

Angiogenesis is a complex process of forming new blood vessels, generally regulated by pro-angiogenic and anti-angiogenic factors (Halder et al., 2018; Wang et al., 2018). However, it is not in dynamic balance in various solid tumors, such as melanoma (Luciano et al., 2021; Parmar and Apte, 2021). Developing a rich vascular network seems vital for melanoma cells during the vertical growth phase, because melanoma cells require lots of nutrients and oxygen to sustain their vertical growth (Pandita et al., 2021). Therefore, angiogenesis is essential for the occurrence and development of melanoma. In 1966, the concept of tumor angiogenesis in melanoma was first proposed by Warren and Shubik (Warren and Shubik, 1966). Since then, anti-angiogenic drugs have been identified as an essential therapeutic measure for treating melanoma (Liu et al., 2022a; Hu et al., 2022; Wohlfeil et al., 2022). These studies suggest that inhibiting angiogenesis will bring new insights into the treatment of melanoma.

In this review, we have elucidated the clinical trials and detailed mechanisms of anti-angiogenesis drugs in melanoma treatment, such as monoclonal antibodies (Bevacizumab, Ramucirumab, Aflibercept, Ontuxizumab), tyrosine kinase inhibitors (Sorafenib, Lenvatinib, Imatinib, Sunitinib, Pazopanib, Axitinib) and human recombinant Endostatin. At the same time, we will further discuss the anti-angiogenic activity of Traditional Chinese herbal medicine. In addition, we will also elucidate potential mechanisms of resistance to anti-angiogenic agents, giving an outlook on the specific targets which would be helpful to the successful therapy of malignant melanoma.

## 2 Mechanism of angiogenesis

**FIGURE 1:**
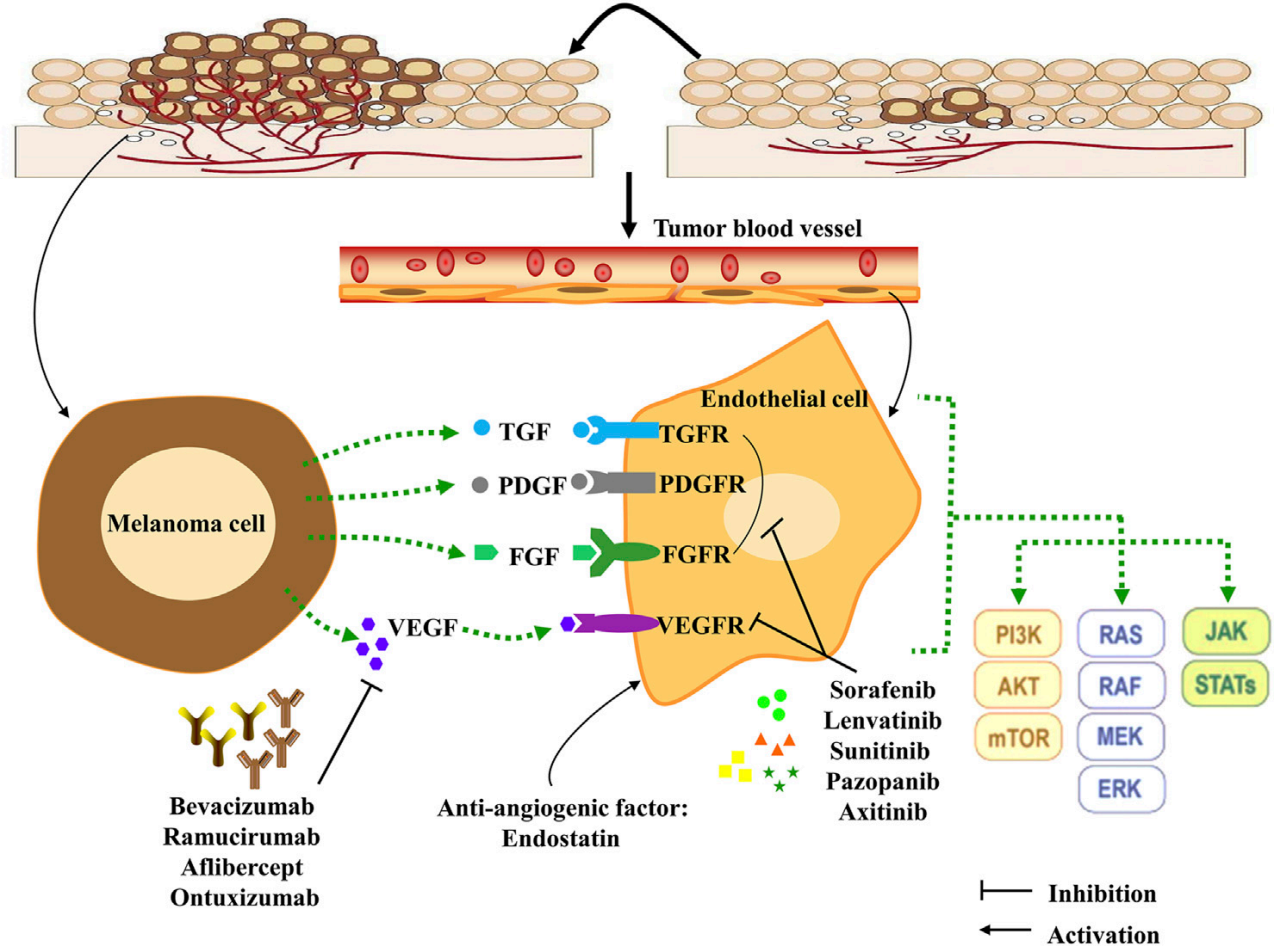
Angiogenesis, angiogenesis signaling pathways and anti-angiogenesis targets in melanoma cells. Pro-angiogenic factors are released by melanoma cells, and can bind receptors expressed on endothelial cells, which leads to initiation of the downstream signaling effects to stimulate melanoma proliferation, metastasis and differentiation. Combining this process with anti-angiogenesis compounds (monoclonal antibodies and TKIs) can effectively inhibit tumor angiogenesis. In addition, the anti-angiogenic factor Endostatin can interact with pro-angiogenic factors to influence angiogenesis in the tumor microenvironment. TKIs: tyrosine kinase inhibitors; TGF: transforming growth factor; TGFR: transforming growth factor receptor; VEGF: Vascular endothelial growth factor; VEGFR: Vascular endothelial growth factor receptor; PDGF: Platelet-derived growth factor; PDGFR: Platelet-derived growth factor receptor; FGF: Fibroblast growth factor; FGFR: Fibroblast growth factor receptor; PI3K: Phosphoinositide 3-kinases; STAT: Signal transducer and activator of transcription protein; JAK: Janus protein tyrosine kinase; MEK: Mitogen-activated protein kinase; mTOR: Mammalian target of rapamycin.

Angiogenesis, forming new blood vessels depending on pre-existing vasculatures (Koo and Kume, 2013), is an essential indicator of tumor proliferation, survival, and distant metastasis in various solid tumors, including melanoma (Cho et al., 2019). Melanoma cells have acquired the ability to induce angiogenesis to meet the increasing nutritional and oxygen needs, especially when cells are in a vertical growth phase of continued proliferation (Straume et al., 1999; Sobierajska et al., 2020). Generally, pro-angiogenic and anti-angiogenic factors are in a dynamic balance (Kazerounian and Lawler, 2018). However, this balance of angiogenesis is often out of control in melanoma. As a result, large amounts of pro-angiogenic factors are release and the expression of the receptors of these factors upregulate in tumor cells. Pro-angiogenic factors will play a dominant role in angiogenesis, leading to the formation of new blood vessels (Rodrigues and Ferraz, 2020). Then, with an adequate supply of nutrients, tumor cells can increase rapidly without control and become more invasive, ultimately leading to metastasis. The growth factors and cytokines are the potential targets for angiogenesis and have been well-studied in melanoma therapy (Figure 1).

Some critical functional enzymes and adhesion factors have been discussed in melanoma, including vascular endothelial growth factor A (VEGF-A), placental growth factor (PlGF), interleukin-8 (IL-8), primary fibroblast growth factor (bFGF), platelet-derived growth factor (PDGF), angiopoietin (Ang), urokinase plasminogen activator (uPA), integrin, and MMPs (Singh et al., 2010; Laurenzana et al., 2017; Zhang et al., 2018a; Lacal and Graziani, 2018; Pekkonen et al., 2018; Zhang et al., 2019; Czarnecka et al., 2020; Ten Voorde et al., 2021). Vascular endothelial growth factor (VEGF), the first described cytokine, stimulates the formation of new blood vessels in tumors (Senger et al., 1983). There are seven types of this gene family, including VEGF-A, VEGF-B, VEGF-C, VEGF-D, VEGF-E, VEGF-F, and placental growth factors (Shibuya, 2011). Accordingly, the VEGF receptor (VEGFR) is a member of the tyrosine kinase receptor family, including five subtypes (VEGFR-1, VEGFR-2, VEGFR-3, NRP-1, and NRP-2) (Zhao et al., 2021). To our knowledge, VEGFRs are the most crucial factors in cancer angiogenesis. For instance, VEGF induces phosphorylation of VEGFR and activates its downstream signaling to enhance vascular expansion and permeability of tumor cells (Fernandez-Cruz et al., 2019). Meanwhile, the other factors (PlGF, IL-8, bFGF, PDGF, Ang, uPA, integrin, and MMPs) are also produced by melanoma cells and endothelial cells. These pro-angiogenic growth factors and cytokines commonly induce their downstream signaling effects through paracrine and autocrine mechanisms. Interestingly, their corresponding receptors are frequently overexpressed by melanoma cells (Peters et al., 2020). Many studies showed that inhibiting pro-angiogenic growth factors and cytokines could attenuate the formation of new blood vessels and inhibit angiogenesis (Muppala et al., 2017; Horvathova et al., 2019; Palanisamy et al., 2019). In this review, we summarized these clinical anti-angiogenic drugs and Traditional Chinese herbal medicine in melanoma (Table 1, Table 2, and Table 3), hoping to improve the clinical effectiveness of anti-angiogenic drugs in treating melanoma.

**TABLE 1:** Summary of clinical stage and ongoing evaluation of anti-angiogenic agents in melanoma.

Class	Agent	Company	Mechanism of action
Monoclonal antibody	Bevacizumab	Roche	VEGF
	Ramucirumab (Cyramza)	Eli Lilly	VEGFR2
	Aflibercept	Bayer AG	Binds to circulating VEGF-A
	Ontuxizumab (MORAB-004)	Morphotek Inc	interferes with Endosialin function
TKIs	Sorafenib	Bayer AG	Receptor tyrosine kinase inhibitor
	Sunitinib	Pfizer	—
	Imatinib	Novartis	BCR/ABL, v-Abl, PDGFR, c-kit
	Lenvatinib	Eisai Co., Ltd.	VEGFR 1–3, FGFR 1–4, PDGFRα, c-KIT, RET
	Pazopanib	Novartis	VEGFR, PDGFRβ, c-Kit, FGFR1, c-Fms
	Axitinib	Pfizer	VEGFR、Kit、PDGFR
Endostatin	—	Simcere	VEGFR

**TABLE 2:** Overview of clinical studies in melanoma cancer of anti-angiogenic therapy.

Drug	Indication	Phase	Pivotal study	End points	Status	Main conclusion
Bevacizumab	melanoma	AVAST-M	ISRCTN 81261306	OS: 64%, no significant difference; DFI: 51% vs. 45%	Recruiting	Adjuvant Bevacizumab can improve DFI, but not OS.
	BRAF mutation melanoma			OS: 63% vs. 55%; DFI: 48% vs. 40%		BRAF mutation status may benefit from Bevacizumab
Bevacizumab + paclitaxel + carboplatin	advanced melanoma	II	NCT02023710	median PFS: 4.8 months vs. 3.0 months; median OS: 13.6 months vs. 9.0 months; ORR: 19.7% vs. 13.2%	Unknown	PFS and OS of Bevacizumab group are better than CPB group alone
Ramucirumab	metastatic melanoma	II	NCT00533702	median PFS: 2.6 months vs. 1.7 months; median OS: 8.7 months vs. 11.1 months; PR: 9 (17.3%) vs. 2 (4.0%); SD: 19 (36.5%) vs. 21 (42.0%)	Completed	Ramucirumab with dacarbazine was associated with an acceptable safety profile in MM patients. PFS appeared greater in combination therapy
Ziv-Aflibercept + Pembrolizumab	naïve melanoma	IB	NCT02298959	ORR: 16.7%	Active, not recruiting	The combination demonstrates an acceptable safety profile and is being studied in sarcoma and anti-PD-1-resistant melanoma
Ziv-Aflibercept + IL-2	inoperable Stage III/IV melanoma	II	NCT00450255	PFS: 6.9 months vs. 2.3 months; OS: no significant difference; ORR: 22% vs. 17%; SD: 65% vs. 48%	Completed	The combination therapy was found to significantly improve PFS.
Ontuxizumab	metastatic melanoma	II	NCT01335009	median PFS: 8.3 weeks; 24-week PFS: 11.4%; median OS: 31.0 weeks; SD: 40.9%	Completed	Ontuxizumab at both doses was well tolerated. Effectiveness of single-agent Ontuxizumab at these doses in melanoma was low
Sorafenib	metastatic uveal melanoma	II	NCT02517736	PFS: 31.2%; OS: 62.5%	Completed	41.4% of patients required dose modifications, and demonstrated no improvement in HRQoL
Sorafenib + chemotherapy (gemcitabine or cisplatin)	metastatic collecting duct carcinoma	II	NCT01762150	OS: 12.5 months; ORR: 30.8%; DCR: 84.6%	Completed	This combination may be a suitable option for patients who have low Eastern Cooperative Oncology Group performance status or less metastatic sites
Lenvatinib	melanoma	I	NCT00121680	PR: 15.6%; SD: 24.7%	Completed	The toxicity, pharmacokinetics, and anti-tumor activity of Lenvatinib are encouraging. Low angiopoietin-1 ratio was correlated with longer PFS.
Lenvatinib + pembrolizumab	unresectable stage III/IV melanoma	II	NCT03776136	ORR: 21.4%; median PFS: 4.2 months; OS: 14.0 months	Active, not recruiting	Lenvatinib plus pembrolizumab as a potential regimen for this population of high unmet need
Imatinib	c-Kit mutations melanoma	II	NCT00881049	median PFS: 4.5 months (mucosal), 2.7 (acral), and 5.0 (unknown-primary); Median OS: 18.0 months (mucosal), 21.8 (acral), 11.5 (unknown-primary)	Completed	KIT-alterations tend to be sensitive to Imatinib
Sunitinib	acral and mucosal melanomas	II	NCT00577382	2-month PF: 52%; DCR: 44%	Completed	The activity of Sunitinib was not dependent on the presence of a KIT mutation. However, the medication was poorly tolerated, and there were no prolonged responses
Pazopanib + paclitaxel	metastatic melanoma	II	NCT01107665	6-month PFS: 8 months; median OS: 12.7 months	Completed	This combination was well-tolerated and demonstrated significant activity
Axitinib	recurrent advanced melanoma	II	NCT03383237	median PFS: 4.0 months; median OS: 12.0 months; DCR: 86.7%	Unknown	As a second or above-line therapy in patients with malignant melanoma. The toxicity was manageable
Axitinib + toripalimab	advanced melanoma	IB	NCT04640545	ORR: 48.3%; median PFS: 7.5 months; TRAEs: 97%	Recruiting	The combination was tolerable and showed promising anti-melanoma activity
Endostatin + chemotherapy	advanced or recurrent mucosal melanomas	II	NCT04699214	PFS: 4.9 months; OS: 15.3 months	Recruiting	This combination was efective and safe. High LMR was correlated with favorable PFS and OS in this patient population

**TABLE 3:** Summary of evaluation of Traditional Chinese herbal medicine in melanoma.

Compound	Source	Mechanism of action
Betulinic Acid	Plane and birch trees	Autophagy, HIF-1/VEGF-FAK signaling pathway
Genistein	Soybean	blocks PGE2
apigenin	Vegetables, fruits, celery and parsley	TNF-α, PI3K/Akt/mTOR signaling pathway
Jatrorrhizine	Coptis Chinensis	Interferes the expression of VE-cadherin
Berberine	Coptis Chinensis	Suppresses pro-angiogenic factors
Capsaicin	Chili	Autophagy, the tNOX-SIRT1 axis
Silymarin	Silybum marianum	Angiogenic biomarkers
Honokiol	Magnolia tree	Hypoxia-inducible-factor, pro-angiogenic genes
Parthenolide	Michelia champaca L	NF-кB/AP-1/VEGF signaling pathway
Cryptotanshinone	Salvia miltiorrhiza	PI3K/Akt/mTOR signaling pathway, MMP/TIMP system and HIF-1α

## 3 Anti-angiogenic agents in melanoma

### 3.1 Monoclonal antibody

#### 3.1.1 Bevacizumab

Bevacizumab, a humanized VEGF monoclonal antibody, is the leading anti-angiogenic agent for clinical use in advanced melanoma (Corrie et al., 2018). It shows anti-tumor effects by preventing the binding of VEGF with its receptors and inhibiting the growth of endothelial cells and vessel formation (Presta et al., 1997). To evaluate the effects of Bevacizumab on patients with melanoma, 1344 patients (median age 56 years) who had resected cutaneous melanoma were recruited (Corrie et al., 2018). They were randomized into the adjuvant Bevacizumab (7.5 mg/kg intravenous every 3 weeks for 1 year) group and the standard observation group. Results showed that the overall survival (OS) at 5 years of the two groups was 64%. However, compared with the observation group, the disease-free interval (DFI) of the Bevacizumab group was 51%, implying that the DFI was improved in the Bevacizumab group. This clinical research also showed that patients with BRAF mutations tended to have poorer OS without Bevacizumab treatment (Corrie et al., 2018). Moreover, a phase II study assessed the activity of Bevacizumab in combination with paclitaxel and carboplatin in patients with advanced melanoma (Yan et al., 2021). 114 patients were enrolled in this research, and patients were randomly assigned to a CPB (carboplatin + paclitaxel + Bevacizumab) group and a CP (carboplatin + paclitaxel) group. The median progression-free survival (PFS) in the CPB group was 4.8 months, which was longer than that in the CP group (3.0 months). The overall response rate (ORR) of the two groups was 19.7% (CPB) and 13.2% (CP), respectively. Meanwhile, the median OS in the CPB group (13.6 months) was also significantly longer than in the CP group (9.0 months). A phase III trial was undertaken to evaluate the serum vitamin D in patients with resected stage IIB–IIIB melanoma after Bevacizumab treatment (Lipplaa et al., 2018). Patients with resected stage IIB-C and IIIA-C melanoma randomly receiving Bevacizumab (7.5 mg/kg every 3 weeks) or observation. One year later, vitamin D levels of patients did not predict prognostic markers DFI (HR = 0.98 per 10 nmol/L increase) or OS (HR = 0.96 per 10 nmol/L increase). Interestingly, longer DFI was observed in stage II melanoma patients after Bevacizumab treatment with higher vitamin D levels. Further exploration is warranted in the future.

#### 3.1.2 Ramucirumab

Ramucirumab is a fully humanized anti-VEGF-2 monoclonal antibody that inhibits tumor growth and angiogenesis (Goode and Smyth, 2016; Jagiela et al., 2021). The safety, tolerability, and effectiveness of Ramucirumab when used alone or in combination with dacarbazine in patients with metastatic melanoma were assessed (Carvajal et al., 2014). In a phase II study, 106 patients with metastatic melanoma were enrolled from 14 centers in America. Patients received Ramucirumab at a dose of 10 mg/kg every 3 weeks (q3w) or Ramucirumab 10 mg/kg plus dacarbazine 1000 mg/m^2^ intravenously q3w. The Median PFS in the Ramucirumab group was 2.6 months compared with 1.7 months in the combination group. The Median OS of the Ramucirumab group was 8.7 months, and 11.1 months of combination therapy, respectively. (Carvajal et al., 2014). The combination group (Ramucirumab plus dacarbazine) showed safety at grade 3/4 toxicities. In conclusion, the preliminary effectiveness of Ramucirumab plus dacarbazine demonstrated the importance of VEGFR-2 inhibition in treating metastatic melanoma. A phase Ia/Ib study of LY3300054 (a new programmed cell death ligand 1 (PD-L1) inhibitor) as monotherapy or in combination with Ramucirumab, neratinib (a type II MET kinase inhibitor) or abemaciclib in patients with solid tumors was conducted. As a result, LY3300054 was well tolerated when administered alone or concurrently with Ramucirumab. No adverse events associated with the combination were observed. Durable clinical effects were observed in LY3300054 dose (phase Ia) as monotherapy or combined with Ramucirumab (Patnaik et al., 2021).

#### 3.1.3 Aflibercept

Aflibercept (VEGF Trap) is a selective humanized IgG1 monoclonal antibody, which can block the interaction of VEGF and its receptors (VEGFR1 and VEGFR2). Angiogenesis could cause immune suppressions in multiple solid tumors (Li et al., 2021). To investigate the effects of the therapy (Ziv-Aflibercept + pembrolizumab) on melanoma, a phase IB trial was conducted (Rahma et al., 2022). Ziv-Aflibercept (2–4 mg/kg) or pembrolizumab (2 mg/kg) was administered intravenously every 2 weeks. No dose-limiting toxicities were observed at the initial dose level, and 2 of 33 patients had a complete response, and 1 had a partial response. The combination group showed an acceptable safety profile with anti-tumor activity in melanoma. The study is currently being carried out in patients with anti-PD-1-resistant melanoma (NCT02298959) (Rahma et al., 2022). The VEGF family takes a pivotal part in mediating tumor and lymph angiogenesis as well as the innate and adaptive immunities of the host (Fagiani et al., 2016; Boudria et al., 2019; Gloger et al., 2020; Menzel et al., 2020). As reported, Interleukin-2 (IL-2), a growth factor for T and NK cells, plays a significant role in melanoma (Pretto et al., 2014; Lee et al., 2016). To investigate the effect of Aflibercept with IL-2 on metastatic melanoma, a phase II study was implemented (Tarhini et al., 2018). 89 patients were enrolled and randomly divided into the combination group (Aflibercept + IL-2) or the IL-2 isolated group. Results showed that the PFS of the combination group was 6.9 months, and that of the IL-2 alone group was 2.3 months. Although there were no significant differences in OS between the two groups, the PFS of IL-2 and Ziv-Aflibercept group significantly improved compared with IL-2 alone group, suggesting that anti-VEGF combined with immunosuppressive agents might be an excellent therapeutic option for patients with melanoma.

#### 3.1.4 Ontuxizumab

Ontuxizumab (MORAB-004), the first monoclonal antibody that interferes with the function of Endosialin, plays an essential role in tumor growth and angiogenesis. The clinical activity and tolerability of Ontuxizumab were evaluated in a phase II study (D’Angelo et al., 2018). In this trial, patients with metastatic melanoma who had received at least one prior systemic treatment received Ontuxizumab weekly at 2 or 4 mg/kg. The median PFS was 8.3 weeks in the Ontuxizumab group. Moreover, the overall grade 1 or 2 adverse events were nausea (36.8%), headache (55.3%), chills (42.1%), and fatigue (48.7%). In summary, the effectiveness of Ontuxizumab (2 or 4 mg/kg) in melanoma was poor. Clinical trials aimed at evaluating the effectiveness of Ontuxizumab alone, and in combination with other active drug may yield better results.

### 3.2 Tyrosine kinase inhibitors

Tyrosine kinase inhibitors (TKIs) are involved in tumorigenesis and progression, which aims to inhibits the catalytic function of kinases and then blocks the activation of downstream signaling cascade (Bellantoni and Wagner, 2021; Salmaso et al., 2021). In recent years, TKIs have identified as critical targets for drug discovery (Choi et al., 2021; Mohammadi and Gelderblom, 2021; Yang et al., 2022). Summary of clinical stage and ongoing evaluation of TKIs might contribute to a comprehensive understanding of TKIs therapies in cancer.

#### 3.2.1 Sorafenib

Sorafenib is a raf kinase inhibitor, which can also inhibit the tyrosine kinase activity of various receptors, including VGFR-2, VEGF-3, PDGF-β, KIT, FLT-3, and other receptors. Sorafenib has dual anti-tumor effects (Ortega-Muelas et al., 2021), which can not only directly inhibit the proliferation of tumor cells by mediating RAF/MEK/ERK pathway but also cut off the nutrition of tumor cells through inhibiting the formation of new blood vessels (Spirli et al., 2012). To evaluate the safety, effectiveness, health-related quality of life (HRQoL), and the non-progression rate of Sorafenib (800 mg per day), a multicenter, single-arm phase II trial was conducted in patients with metastatic uveal melanoma. (Mouriaux et al., 2016). After 24 weeks of oral administration, the PFS and OS in the Sorafenib group were 31.2% and 62.5%, respectively. However, 41.4% of patients required dose adjustment due to toxicity and without improvement of HRQoL. Simultaneously, to evaluate the safety and effectiveness of Sorafenib plus chemotherapy (gemcitabine or cisplatin) in metastatic melanoma patients with collecting duct carcinoma, a randomized, single-arm, and multicenter study was carried out (Sheng et al., 2018). The data showed the median OS in the combination group (Sorafenib plus chemotherapy) was about 12.5 months, and the ORR was 30.8%. Delightfully, the PFS for Sorafenib plus chemotherapy was improved in metastatic melanoma patients with CDC.

#### 3.2.2 Lenvatinib

Lenvatinib (E7080) is an oral multiple tyrosine kinase inhibitor (TKI) that shows effects on VEGFR1-3, FGFR1-4, PDGFR, and KIT to inhibit tumor angiogenesis (Okamoto et al., 2013). Moreover, Lenvatinib inhibits human umbilical vein endothelial cell proliferation and tube formations to reduce tumor growth (Capozzi et al., 2019; Iwasa et al., 2020). In a phase I trial, the safety and clinical effectiveness of Lenvatinib were assessed in patients (*n* = 77) with melanoma (Hong et al., 2015). 18 patients received Lenvatinib at a dose of .1–3.2 mg twice a day (BID) (7 days on, 7 days off), while 33 patients received it at an amount of 3.2–12 mg BID and the dose of 10 mg BID in 26 patients, respectively. Preliminary results from this phase I trial shows that Lenvatinib had partial clinical response of 15.6% with a stable disease (SD) ≥23 weeks. The authors also found the dose-limiting toxicities of Lenvatinib included fatigue, hypertension, and proteinuria (Hong et al., 2015). Besides, a decrease in the angiopoietin-1 ratio was considered a significant factor associated with prolonged PFS in melanoma patients. At the same time, angiogenesis and apoptosis-related biomarkers were related to PFS in melanoma patients treated with Lenvatinib. Subsequently, a multicenter, open-label phase Ib/II study (Arance et al., 2022) was taken in melanoma (ClinicalTrials.gov identifier: NCT03776136). Briefly, 103 patients with melanoma were enrolled. Preliminary results showed that the median study follow-up was 15.3 months, and ORR in the total population was 21.4%. The adverse events occurred in 47 (45.6%) patients in Grades 3–5 (Arance et al., 2022). Accordingly, the anti-angiogenic therapy combined with immunotherapy displays promising anti-tumor activities and expected safety profiles in patients with melanoma.

#### 3.2.3 Imatinib

A successful TKI, Imatinib, demonstrates its anti-angiogenic activity against multiple targets, including v-Abl, c-Kit, and PDGFR (Knol et al., 2015). Recent reports showed that c-Kit mutations were more common in acral and mucosal melanomas (Knol et al., 2015; Sabbah et al., 2021). In a study, 130 KIT-altered melanoma patients were pooled from five medical centers (Jung et al., 2022). Mucosal melanoma was associated with high PFS, whereas exon 17 mutations were associated with low PFS. Imatinib has shown significant activity as a therapeutic agent in metastatic melanoma patients harboring aberrations in c-Kit. Thereby, promising prospects of Imatinib in melanoma applications could occur in the future.

#### 3.2.4 Sunitinib

Sunitinib, an oral multi-kinase inhibitor, has been used in melanoma and targets VEGF receptor, KIT receptor, and other receptors (Yeramian et al., 2012). It is well known that the VEGF and KIT are potential targets for alternative therapeutics in malignant melanoma, and they are recognized to play a pivotal role in the pathogenesis and metastasis of melanoma (Graells et al., 2004; Curtin et al., 2006). Based on the part of VEGF and KIT in melanoma, they conducted a phase II trial of Sunitinib for patients with acral and mucosal melanomas (Buchbinder et al., 2015). Patients received 37.5 mg and 50 mg Sunitinib daily. The results showed that the toxicity was acceptable, and the disease control rate was 44%. Fortunately, 20% or more of the patients were alive, and progression-free at 2 months, encouraging the activity of Sunitinib in acral and mucosal melanomas. The results indicated Sunitinib had greater effectiveness in patients with primary KIT exon 9 mutations or wild-type status than in those with direct KIT exon 11 mutations. However, the responses of Sunitinib in patients with non-KIT-mutated indicated Sunitinib might have other targets associated with melanoma growth. The tolerance of Sunitinib was poor, and no lengthy response was observed in patients. With the multi-target features, a combination of Sunitinib and other inhibitors might provide a considerable promise in the future.

#### 3.2.5 Pazopanib

Pazopanib is an oral TKI that binds to VEGFR1-3, c-KIT, PDGFR-α, and PDGFR-β, which are often abnormally activated during tumorigenesis (Goh et al., 2010). A clinical trial using Pazopanib combined with various cytotoxic chemotherapies was investigated in BRAF wild-type metastatic melanoma patients (Fruehauf et al., 2018). 60 patients were included in this study and received Pazopanib and paclitaxel. The final dates displayed that the combination of the Pazopanib plus paclitaxel was well-tolerated, and the significant activity was close to the current first-line therapy for metastatic melanoma. Moreover, the immunological events and metabolic responses induced by Pazopanib plus paclitaxel was evaluated in a study. 90 patients received Pazopanib/paclitaxel, Pazopanib was given 400 mg, BID. Paclitaxel was given 150 mg/m2 body surface. Interestingly, they observed that melanoma cells could be rescued by M2 macrophages after Pazopanib treatment (Thurneysen et al., 2016). Thus, therapies that inhibit tumor-associated macrophages might be feasible and have potential for melanoma patients.

#### 3.2.6 Axitinib

Axitinib is a TKI that targets VEGFR-1, VEGFR-2, c-SRC, Kit, and RET (Roviello et al., 2016). Preclinical studies had demonstrated the vital role of the VEGF signaling pathway in melanoma (Peng et al., 2016). Importantly, Yuan et al. (2022) conducted a single-center, single-arm phase II trial in patients with advanced recurrent melanoma. Patients orally received Axitinib daily. Regarding the toxicity, results elaborated that Axitinib was well tolerated, and the observed toxicity levels were mild and manageable. In summary, Axitinib might play a vital role in metastatic melanoma, and further investigations of Axitinib alone or in combination with chemotherapy should be taken.

Mucosal Melanoma is a severe natural disease (Lian et al., 2017). Unfortunately, due to the rarity, there are no well-established therapeutic guidelines for treating mucosal melanoma (Yi et al., 2011; Lian et al., 2017). In the past decade, the application of targeted therapies and immunotherapies has brought light in metastatic cutaneous melanoma treatment (Kaufman et al., 2018). Xinan Sheng et al. (2019) had reported the safety and effectiveness of toripalimab and Axitinib in patients with advanced melanoma. They found high response rates (48.3% ORR) and prolonged median PFS for toripalimab and Axitinib. Moreover, tumor mutational burden (TMB) and PD-L1 expression were related to higher ORR, consistent with previous reports (Hellmann et al., 2018). Interestingly, three published signatures (angiogenesis signatures, inflammation signatures, and interferon-gamma signatures) for clinical outcomes were also discussed in their study (Hellmann et al., 2018), which might be relevant biomarkers for immuno-oncology plus VEGF therapy. In summary, Axitinib combined with toripalimab could be a promising option for mucosal melanoma treatment. Subsequently, a phase III study should be validated among non-Asian patients in the future.

### 3.3 Endostatin

Endostatin (20-kDa), a potent endogenous angiogenesis inhibitor, is the C-terminal fragment of type XVIII collagen (O'Reilly et al., 1997). Since 1997, Endostatin has shown anti-angiogenic effects on endothelial cells (Alahuhta et al., 2015; Jia et al., 2017; Lamattina et al., 2019; Wang et al., 2021a; Shin et al., 2021; Zhu et al., 2022). In 2005, Endostatin was approved by the Food and Drug Administration of China to treat non-small-cell lung cancer (NSCLC) (Wang et al., 2005; Han et al., 2011). However, the clinical effectiveness of Endostatin is controversial, which needs further investigation in the treatment of patients with metastatic melanoma. A real-world study was designed to evidence the effectiveness and safety of Endostatin plus chemotherapy for treating patients with metastatic melanoma (Zhang et al., 2022). In this trial, 43 patients with advanced or recurrent mucosal melanoma were recruited from Fudan University Shanghai Cancer Center (April 2017 and August 2020). They were randomly assigned to the two arms (dacarbazine plus cisplatin arm, temozolomide plus cisplatin). Simultaneously, patients in the two arms received a placebo or Endostatin (105 mg/m) intravenously for 168 h. At the end of this trial, the PFS and OS were 4.9 and 15.3 months, respectively. Endostatin plus chemotherapy represented well tolerability and a manageable toxicity profile (Zhang et al., 2022). Overall, Endostatin provided a novel option for anti-angiogenic treatment.

Endostatin has been recently identified as a prognostic biomarker for patients with metastatic melanoma (Nyakas et al., 2019). Many studies showed Endostatin levels were closely associated with aggressive phenotypes or poor outcomes in various malignancies (Alahuhta et al., 2015; Chen et al., 2018; Zamaratskaia et al., 2020; Zhang et al., 2021a; Zhu et al., 2022), such as metastatic melanoma (Fukuda et al., 2011; Liang et al., 2018; Zhang et al., 2022). A phase IV study demonstrated Endostatin could influence melanoma invasion by regulating T Cell activation (Nyakas et al., 2019). As a prognostic biomarker for metastatic melanoma patients, Endostatin might be helpful in selecting patients for anti-angiogenic therapy.

### 3.4 Traditional Chinese herbal medicine

In recent years, anti-cancer compounds extracted from Traditional Chinese Medicine (TCM) have become a research hotspot. Several studies have reported significant anti-angiogenic activities of these compounds in melanoma, the underlying mechanisms of which are still being studied. Firstly, Betulinic Acid (BA), an extract from the plane and birch trees, has shown anti-angiogenic effects in melanoma. An *in vitro* study indicates that BA significantly inhibits the proliferation of melanoma cell lines (Wroblewska-Luczka et al., 2022). Interestingly, the combination of BA with paclitaxel or docetaxel indicates ideal drug-drug synergy interactions (Wroblewska-Luczka et al., 2022). Another study reports that BA demonstrated inhibitory effects on A375 melanoma cells *via* mitochondrial apoptosis and glycolysis pathway (Coricovac et al., 2021). Next, genistein (GS), derived from the soybean, is a powerful anti-angiogenic agent in melanoma. It is reported that GS shows an effect on the Prostaglandin E2 (PGE2) pathway, which has been proven as essential for its anti-melanoma activity. Furthermore, the overexpression of IL-8 could be induced by GS through one of the PGE2 receptors (EP3) in melanoma cells (Venza et al., 2018). Simultaneously, apigenin, which is a naturally occurring flavonoid in vegetables, fruits, celery, and parsley, could inhibit the proliferation and angiogenesis of melanoma cells by suppressing the secretion of TNF-α and influencing PI3K/Akt/mTOR signaling pathway (Li et al., 2019b; Ghitu et al., 2019; Ghitu et al., 2021). Furthermore, jatrorrhizine hydrochloride (JH), a component of Coptis Chinensis, shows anti-metastatic and anti-proliferation effects on C8161 human melanoma cells. Mechanistic studies showed that JH induced G0/G1 cell cycle arrest in C8161 tumor cells. Moreover, JH reduced the neovascularization of C8161 cells and disturbed the expression of VE-cadherin, suggesting that JH is a new potential anti-melanoma drug candidate (Liu et al., 2013). To explore other effective strategies for treating melanoma, Vaid M et al. assessed the effects of Silymarin (an extract of Silybum marianum) on melanoma cells. The data showed the therapeutic effect of Silymarin was associated with angiogenic biomarkers (Vaid et al., 2015). Besides, honokiol, a compound isolated from the Magnolia tree, has a therapeutic impact on skin cancer (Leeman-Neill et al., 2010). More interestingly, the anti-angiogenic functions of some of these TMCs are strongly associated with hypoxia-inducible-factor (HIF) and other pro-angiogenic genes (Vavilala et al., 2012). For instance, Parthenolide (PT), an active component of the medicinal herb Feverfew, exhibits an anti-angiogenic effect by regulating the NF-кB/AP-1/VEGF signaling pathway, encouraging a promising agent for melanoma treatments (Talib and Al Kury, 2018; Tian et al., 2020). Particularly, Cryptotanshinone (CPT), isolates from Salvia miltiorrhiza, takes a crucial role in angiogenesis-related diseases. Zhang et al. (2018b) reported that CPT prevented the growth and metastasis of colon cancer cells *via* modulating PI3K/Akt/mTOR signaling, MMP/TIMP system, and HIF-1α nuclear translocation (Zhu et al., 2016). However, the clinical use of TCM still has severe limitations, which often reduce their therapeutic effectiveness. Consequently, it is urgent to improve their anti-tumor activities in patients.

## 4 Mechanisms of resistance

Angiogenesis plays a crucial role in regulating vital functions of tumor cells, including tumor growth, proliferation, and metastasis (Papaevangelou et al., 2018; Zhang et al., 2021b; Rampino et al., 2021). In recent years, multiple anti-angiogenic agents have been developed to treat melanoma (Huang et al., 2021a; Micheli et al., 2021). However, due to acquired resistance (Jimenez-Valerio and Casanovas, 2017; Pozas et al., 2019; Kuczynski and Reynolds, 2020; Watanabe, 2021), anti-angiogenic agents are limited, including vascular mimicry (VM), vascular co-option, metabolic symbiosis, upregulation of alternative pathways, and recruitment of tumor stromal cells. Moreover, autophagy, a highly mediated adaptive process of cancers, has been implicated in perturbing resistance to anti-angiogenic therapy. Firstly, a recent study determined the inhibitory effect of the BRAFV600E inhibitor vemurafenib on VM in invasive melanoma cells. As a result, vemurafenib failed to inhibit the VM ability of A375 melanoma cells *in vitro* (Andreucci et al., 2022). Another critical factor, vessel co-option, seems to play an essential role in mediating resistance to anti-angiogenic drugs (Kuczynski and Reynolds, 2020). For instance, several studies have shown vessel co-option is associated with primary melanoma and organ (brain, lung, and liver) metastases of melanoma (Lugassy et al., 2014; Szabo et al., 2015; Bentolila et al., 2016; Barnhill et al., 2018; Rodewald et al., 2019), which may be an essential factor for poor clinical effectiveness of anti-angiogenic drugs. Secondly, in terms of the resistance mechanism to anti-angiogenic therapy, metabolic symbiosis is also reported in both experimental and clinical studies (Jimenez-Valerio et al., 2016; Sebestyen et al., 2021). Both OXPHOS and glycolysis (metabolic symbiosis) have been identified to be critical for metabolic plasticity in melanoma, driving acquired resistance to anti-angiogenic chemotherapy (Kumar et al., 2021). Furthermore, the abnormal upregulation of both OXPHOS and glycolysis is significant for melanoma progression (Feichtinger et al., 2018; Ruocco et al., 2019). Thus, inhibition of glycolysis may be a promising strategy to overcome Bevacizumab resistance (Eriksson et al., 2018). Although anti-VEGF therapy is available, drug resistance often occurs, and malignant tumor patients are not always responsive. This acquired resistance to anti-VEGF treatment is involved in other angiogenic pathways, compensating for the inhibiting effects on cancer cells (Li et al., 2014; Choi et al., 2015; Mahdi et al., 2019; Yin et al., 2019). Consequently, combined with multitargeted inhibitors can refrain angiogenesis more efficiently than monotherapy therapy.

Drug resistance and tumor angiogenesis are affected by the tumor microenvironment (TME) (Maacha et al., 2019), which is composed of stromal cells, immune cells, cancer stem cells (CSCs), blood vessels, tumor cells, lymphatic vessels, and extracellular matrix (ECM) (Liu et al., 2022b). TME is a complex network of tumor cells and surrounding components, where various associated cells and components communicate to regulate tumor growth (De Palma et al., 2017; Wang et al., 2021b). Tumor cells generally prefer a hypoxic environment (Huang et al., 2021b). Unfortunately, long-term use of anti-angiogenic drugs often aggravates hypoxia. Hypoxia-induced upregulation of hypoxia-inducible factor (HIF)-1a can induce the differentiation of tumor cells into CSCs, which is also the main contribution to drug-resistance of anti-angiogenic therapy (Zheng et al., 2018; Jiang et al., 2020). As the host immune system is often disrupted in cancer patients, the increased number of immunosuppressive cells, such as tumor-mass associated macrophages (TAMs), T-regs, and myeloid-derived suppressor cells (MDSCs), will be is responsible for an unfavorable prognosis of cancer treatment (Hosseini et al., 2019; Qi et al., 2020). For this reason, stromal cells, a critical surrounding components of tumor cells, might act as potential therapeutic targets for tumor cells (Licarete et al., 2020). As discussed above, resistance mechanisms of anti-angiogenic therapy have been elucidated in cancer, most of which occurred at the later stage of tumor progression. Different from those, autophagy seems to be the first defense process that appears at the cellular level without extracellular matrix (ECM) or tissue remodeling and needs to understand better (Chandra et al., 2020; Jena et al., 2021; Wen et al., 2022). Additional studies have revealed autophagy as a resistance mechanism and could enhance anti-angiogenic therapeutic effects (Huang et al., 2018; Zhao et al., 2018; Malhotra et al., 2019). However, whether early or late autophagy inhibitors will overcome the resistance of anti-angiogenic therapy is imperative to determine.

## 5 Summary and future directions

Anti-angiogenic therapy for tumors has achieved specific clinical efficacies (Qin et al., 2019; Tong et al., 2019; Liu et al., 2021a; Yetkin-Arik et al., 2021; Choi et al., 2022), and mainly manifests the improvements of PFS, which is consistent with the fact that angiogenesis is a marker of cancer. The in-depth studies on tumor angiogenesis will become a hot topic in tumor research. In this review, we discussed the critical roles of angiogenesis in melanoma growth and progression. Angiogenesis is a highly complex and dynamic process mediated by pro-angiogenic and angiogenesis inhibitory factors, which is the basis of anti-angiogenesis resistance (Tiwari et al., 2018; Cho et al., 2019). We also discussed the limitations of anti-angiogenic therapies, challenges, safety, predictive biomarkers, and future directions. Simultaneously, we explained Traditional Chinese herbal medicine as a vital anti-angiogenesis option in melanoma therapy.

Angiogenesis provides an essential target for multiple therapeutic agents, including Bevacizumab. VEGF is overexpressed and associated with prognosis in melanoma patients (Liu et al., 2021b). However, anti-angiogenic therapy is not as effective as initially hoped, and drug resistance always occurs in patients with melanoma, especially in those treated with Bevacizumab monotherapy (Jour et al., 2016; Zhang et al., 2020). Combinational therapies might be advantageous as they have multi-mechanisms targeting individual ligands and receptors to avoid resistance. For instance, activating mutations at V600 of the BRAF gene is common in several cancers (Halle and Johnson, 2021), including approximately 50% of melanoma (Gutierrez-Castaneda et al., 2020). Therefore, BRAF/MEK inhibitors have been developed to treat patients with BRAF-mutant melanoma. Moreover, due to the development of drug resistance and tumor recurrence, patients with BRAF-mutant melanoma have a short response time to BRAF/MEK inhibitors (Kakadia et al., 2018). Fortunately, angiogenesis inhibitors might be suitable for BRAF-mutant melanoma patients with acquired resistance to BRAF/MEK inhibitors (Amann et al., 2017; Martin et al., 2018; Atzori et al., 2020). Besides, there is a rationale for anti-angiogenic drugs combined with PARP inhibitors. Combining PARP inhibitors with anti-angiogenic drugs could provide synergetic benefits to patients with solid tumors (Tentori et al., 2007; Ledermann, 2017; Russo and Giavazzi, 2018; Sachdev et al., 2019; Smith and Pothuri, 2022). Mechanistically, PARP1 is associated with the stabilizing of HIF-1α (Hulse et al., 2018), which plays a vital role in melanocyte transformation and represents an essential feature in malignant tumor growth, including melanoma (Malekan et al., 2021).

The therapy of malignant tumors has opened the era of immunotherapy (Wang et al., 2021c; Zhang and Xiao, 2021; Zimmermannova et al., 2021). Both immune checkpoint inhibitors (CPIs) and anti-angiogenic agents have been widely used in melanoma treatment. Numerous trials assessing the effectiveness and safety of anti-angiogenic agents plus CPIs have been taken. The combined strategy is frequent in clinical trials for patients with unresectable stage III or IV melanoma. Most importantly, the combined approaches of Bevacizumab and ipilimumab might synergistically increase the infiltration of CD163+ dendritic macrophages and CD8^+^ T Cells *via* tumor vasculatures (Hodi et al., 2014; Ott et al., 2015). Another combination study of Lenvatinib and pembrolizumab has shown manageable safety and promising anti-tumor activity in patients with melanoma (Taylor et al., 2020). Overall, immunotherapy combined with anti-angiogenic agents does bring survival benefits to patients. Unfortunately, there are currently no drugs that can successfully target both immune systems and blood vessels. Hence few of these drugs could improve tumor progression effectively without adverse reactions or drug resistance. We hope to develop specific targeted inhibitors that affect both immunity and blood vessels to achieve satisfactory anti-tumor effects and prolong the survival of patients in the future, such as evaluating the potential synergistic effects of combined immunotherapy with VEGF inhibitors.

Although modulating angiogenesis appears to be a potential strategy for melanoma treatment, vascular disrupting agents have poor selectivity to distinguish tumor blood vessels from normal blood vessels, thus limiting their ability to suppress tumor growth (Mukherjee et al., 2020; Smolarczyk et al., 2021). The development of drugs that selectively target tumor blood vessels and angiogenesis drivers may be a direction in the future. Furthermore, vascular normalization could not only improve the delivery ratios of drugs but also enhance the therapeutic effects of combination therapies (Zhou and Gallo, 2009). Interestingly, anti-angiogenic treatments could affect tumor vessel normalization, which has synergistic effects when combined with radiotherapy, chemotherapy, immunotherapy strategies, and other therapeutic methods (Viallard and Larrivee, 2017; Liu et al., 2021c; Wang et al., 2021d; Une et al., 2021). However, the main problem of anti-angiogenic therapies is how to confirm the optimized time point and suitable dose of anti-angiogenic agents, which is particularly relevant to expand the vascular normalization window and obtaining the most extended survival time of cancer patients. In addition, prognostic markers, including PFS and PSA responses, did not display their suitability in determining the activity of angiogenesis inhibitors, calling for more energy in this setting. We highlighted the understanding of the molecular pathways that contributed to the development and progression of melanoma, as well as the specific molecular markers and predictors of each melanoma subtype. However, it is debatable whether or not anti-angiogenic therapy should be used as preoperative or perioperative treatment, which needs to be further explored in cancers. Although the efficacies of anti-angiogenic drugs need to be further improved, anti-angiogenic therapies have become an essential milestone in the history of human cancer treatment. It is expected to enhance the effectiveness of anti-angiogenic drugs by understanding the mechanisms of drug resistance and identifying its reliable predictive markers. In conclusion, the drug resistances, side effects, limited survival advantage, and high cancer recurrence rates highlight the critical need for new targets and strategies for anti-angiogenic therapies.
